# A Rare Case of a 15-Year-Old Boy with Two Accessory Nipples: One in the Forearm and One in the Milk Line

**DOI:** 10.1155/2015/752479

**Published:** 2015-12-10

**Authors:** Alexander J. Tauchen, Essie Kueberuwa, Kenneth Schiffman, Kumaran M. Mudaliar, Shelley S. Noland

**Affiliations:** ^1^Department of Orthopaedic Surgery, Loyola University Medical Center, Maywood, IL 60153, USA; ^2^University of Chicago Medical Center, Department of Surgery, Section of Plastic & Reconstructive Surgery, Chicago, IL 60637, USA; ^3^Department of Pathology, Loyola University Medical Center, Maywood, IL 60153, USA

## Abstract

A 15-year-old male presented for evaluation of a volar forearm mass that he noticed four years before. The mass was not painful and his main concern was cosmesis. The mass was two centimeters in diameter with a pinpoint central sinus and scant drainage. After excision, the pathology report noted pilosebaceous units and smooth muscle bundles, consistent with an accessory nipple. In addition, the patient had another accessory nipple in the “milk line” on his torso. While accessory nipples and breast tissue have been reported in numerous locations throughout the body, this is the first reported case of an accessory nipple on the forearm.

## 1. Introduction

Accessory nipples and accessory breast tissue have been described in various locations throughout the body. This tissue is important to recognize, as it is often a cosmetic concern for the patient but may also undergo both benign and malignant transformation. To our knowledge, this is the first reported case of an accessory nipple on the forearm.

## 2. Case Report

A 15-year-old right hand dominant male presented with a chief complaint of a soft tissue mass on the volar surface of his left forearm. He first noted the mass around age 11. The mass grew slowly between the ages of 11 and 13 before stabilizing in size two years prior to presentation. Initially the mass expressed a thick whitish fluid when squeezed, but at the time of presentation the fluid was thin and the drainage minimal. The mass was not painful and he reported no paresthesias in the hand. He denied any similar masses elsewhere on the body and had no history of malignancy. There was no family history of similar masses. He did not have a history of urinary tract malformation.

**Figure 1:**
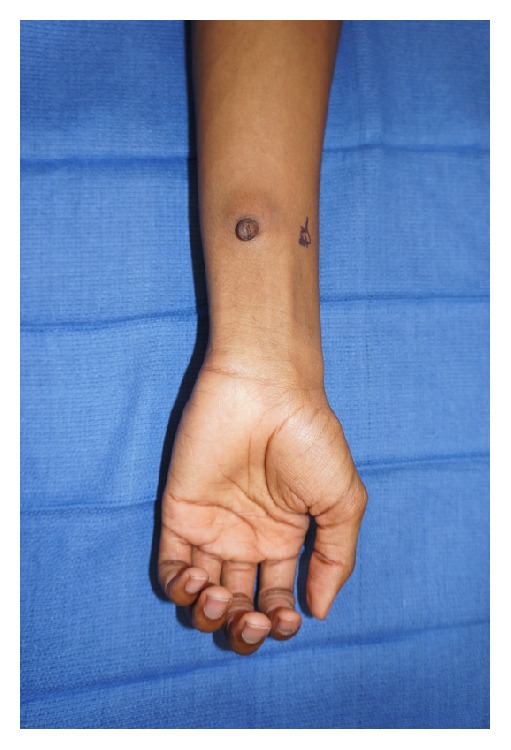
Volar view of forearm.

**Figure 2:**
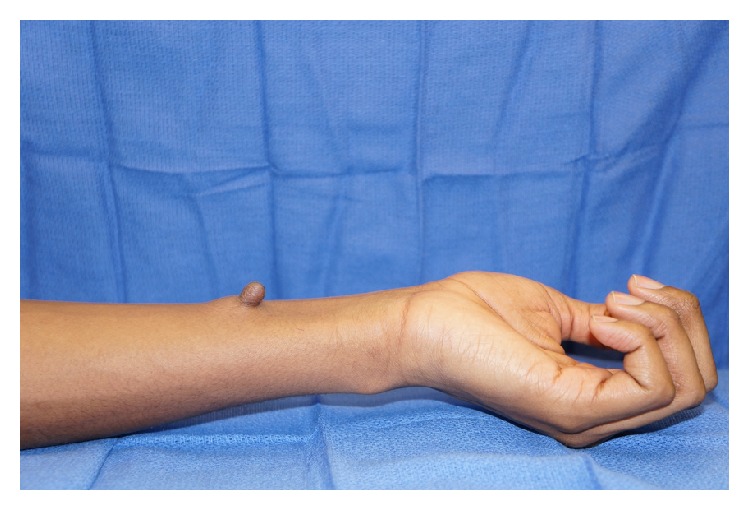
Lateral view of forearm.

**Figure 3:**
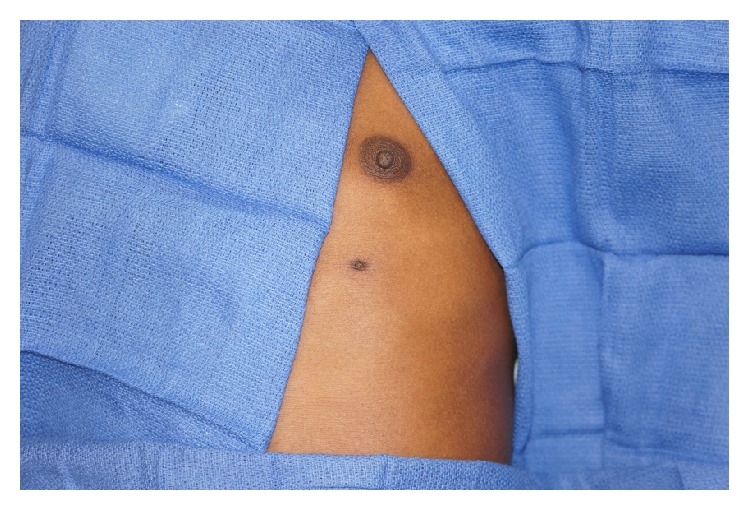
Accessory nipple in “milk line.”

On physical examination, the patient was a well-appearing 15-year-old male in no acute distress. On the volar surface of the left mid forearm, there was a 2.0 × 1.5 cm pedunculated mass with a pinpoint central sinus (Figures 1 and 2). The mass was well-circumscribed, soft, and mobile. There was no surrounding erythema or warmth. A scant amount of thin, white fluid could be expressed from the central sinus. He had normal strength and sensation in the left elbow, wrist, and hand and had a palpable radial pulse. The remainder of his physical exam was remarkable for an accessory nipple on the left chest wall along the vertical “milk line” (Figure 3).

**Figure 4:**
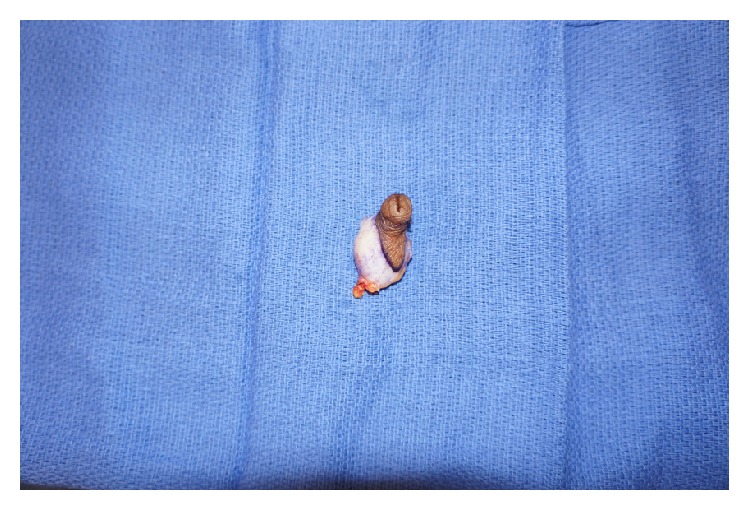
Resected specimen.

Given the prior growth and bothersome appearance, surgical excision of the mass was planned. Prior to surgery, consent for photography was obtained from both the patient and his father. An elliptical longitudinal incision was used to excise the mass and the underlying tissue. The mass was firm and well-circumscribed and had the appearance and texture of breast tissue (Figure 4). The skin was closed using absorbable sutures. A soft dressing was applied and the patient recovered uneventfully.

**Figure 5:**
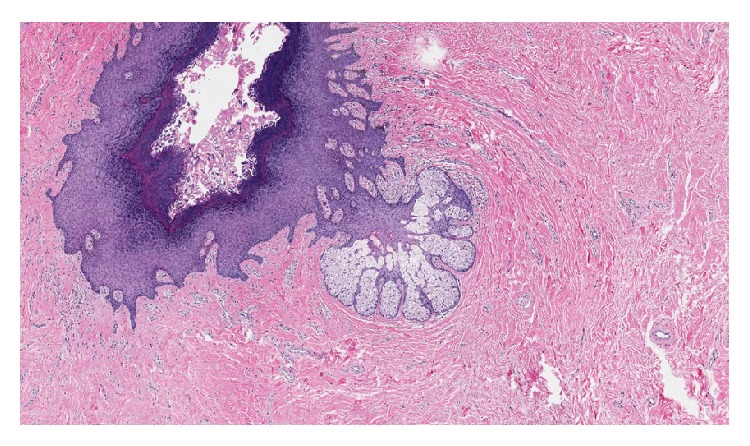
Low power pathology slide (features of accessory nipple including increase in pilosebaceous units and smooth muscle bundles).

**Figure 6:**
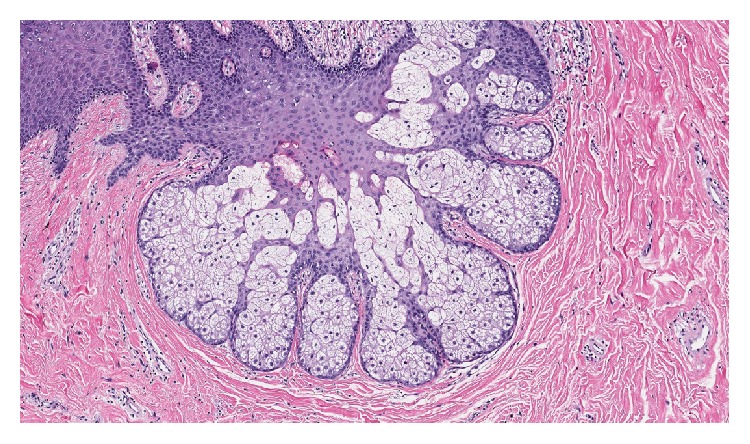
High power pathology slide (sebaceous gland with increase in surrounding smooth muscle bundles).

The soft tissue specimen with an ellipse of skin measuring 2.5 × 1.0 × 0.3 cm and a raised nipple lesion measuring 1.0 × 1.0 × 0.8 cm was sent to pathology for evaluation. The final pathology report described the mass as a fibroepithelial polyp with features of accessory nipple. Pilosebaceous units and smooth muscle bundles were seen but no mammary gland tissue was noted (Figures 5 and 6).

At a two-week follow-up visit, the patient had no complaints and was happy with the appearance of his forearm. The final pathology report was reviewed with the patient and his father and they were made aware that this mass likely represented an accessory nipple.

## 3. Discussion

*Polythelia* is defined as an accessory nipple with or without an areola or accessory breast tissue.* Polymastia* is defined as accessory glandular breast tissue with or without an areola and nipple [1, 2]. The prevalence of accessory breast tissue is reported as 0.22–5.6% [2–5]. About one-third of women presenting with an accessory nipple or breast are symptomatic during menstruation or lactation and are also concerned about cosmesis [4]. Among children specifically, there has been an association of accessory nipples with urinary tract malformations [6].

Accessory nipples have been reported in numerous locations about the body but are most commonly found along the anterior chest wall and abdomen in line with the mammary ridges present during embryological development. This embryonic “milk line” extends from the axilla to the groin. In women, this ectopic breast tissue tends to stay dormant until influenced by sex hormones during puberty or pregnancy [1, 4]. The patient described presented similarly and was asymptomatic until puberty. Other case reports have been published reporting accessory nipples and breast tissue found in the perineum [7], thigh [8, 9], face [10], vulva [11], shoulder [12], and foot [13]. To our knowledge, this is the first reported case of an accessory nipple on the forearm.

Alghamdi and Abdelhadi questioned when excision of accessory breast tissue is necessary [4]. In their series, 233 women were found to have an axillary accessory breast and 66 (28%) underwent surgical excision, mainly due to cosmetic concerns but also due to pain in some cases. In 62% of these cases, the excised specimen was normal breast tissue. There was one case of fibroadenoma and one case of invasive ductal carcinoma in their series. While accessory breast tissue is in an abnormal location, it has the potential to undergo both benign and malignant transformation and must be monitored [4, 14]. The decision to excise the tissue should be made on a case-by-case basis. The patient in our case had no further diagnostic work-up for additional congenital abnormalities.

For each patient presenting with an accessory nipple or breast tissue, it is important to address their main concerns. The patient should be reassured that aside from cosmesis the natural history of the tissue is typically benign. If the concern is mainly cosmetic, they should be advised of the risks and morbidities associated with surgery. In our case, the patient was a teenage male who felt his forearm was unsightly and thus he wanted the mass removed. The pathology was benign and was consistent with an accessory nipple. We believe this to be the first report of accessory nipple located on the forearm.
